# Hinokitiol Inhibits Migration of A549 Lung Cancer Cells via Suppression of MMPs and Induction of Antioxidant Enzymes and Apoptosis

**DOI:** 10.3390/ijms19040939

**Published:** 2018-03-22

**Authors:** Thanasekaran Jayakumar, Chao-Hong Liu, Guan-Yi Wu, Tzu-Yin Lee, Manjunath Manubolu, Cheng-Ying Hsieh, Chih-Hao Yang, Joen-Rong Sheu

**Affiliations:** 1 Graduate Institute of Medical Sciences, College of Medicine, Taipei Medical University, Taipei 110, Taiwan; tjaya_2002@yahoo.co.in (T.J.); y4509@yuanhosp.com.tw (C.-H.L.); m120101037@tmu.edu.tw (G.-Y.W.); d119103001@tmu.edu.tw (T.-Y.L.); 2 Department of Dermatology, Yuan’s General Hospital, Kaohsiung 249, Taiwan; 3 Department of Evolution, Ecology and Organismal Biology, Ohio State University, Columbus, OH 43212, USA; manubolu.1@osu.edu; 4 Department of Pharmacology, School of Medicine, College of Medicine, Taipei Medical University, Taipei 110, Taiwan; hsiehcy@tmu.edu.tw

**Keywords:** A549 cells, hinokitiol, MMPs, p53/Bax, antioxidant enzymes, caspases, migration

## Abstract

Hinokitiol, a natural monoterpenoid from the heartwood of *Calocedrus formosana*, has been reported to have anticancer effects against various cancer cell lines. However, the detailed molecular mechanisms and the inhibiting roles of hinokitiol on adenocarcinoma A549 cells remain to be fully elucidated. Thus, the current study was designed to evaluate the effect of hinokitiol on the migration of human lung adenocarcinoma A549 cells in vitro. The data demonstrates that hinokitiol does not effectively inhibit the viability of A549 cells at up to a 10 µM concentration. When treated with non-toxic doses (1–5 µM) of hinokitiol, the cell migration is markedly suppressed at 5 µM. Hinokitiol significantly reduced p53 expression, followed by attenuation of Bax in A549 cells. A dose-dependent inhibition of activated caspase-9 and -3 was observed in the presence of hinokitiol. An observed increase in protein expression of matrix metalloproteinases (MMPs) -2/-9 in A549 cells was significantly inhibited by hinokitiol. Remarkably, when A549 cells were subjected to hinokitiol (1–5 µM), there was an increase in the activities of antioxidant enzymes catalase (CAT) and superoxide dismutase (SOD) from the reduction in cells. In addition, the incubation of A549 cells with hinokitiol significantly activated the cytochrome c expression, which may be triggered by activation of caspase-9 followed by caspase-3. These observations indicate that hinokitiol inhibited the migration of lung cancer A549 cells through several mechanisms, including the activation of caspases-9 and -3, induction of p53/Bax and antioxidant CAT and SOD, and reduction of MMP-2 and -9 activities. It also induces cytochrome c expression. These findings demonstrate a new therapeutic potential for hinokitiol in lung cancer chemoprevention.

## 1. Introduction

Lung adenocarcinoma is one of the most severe cancers related to deaths globally, and it is becoming more common in numerous countries. Almost 40% of lung cancers are adenocarcinomas, which are non-small cell lung cancers with a normally poor diagnosis and extremely high rate of latent metastasis [1]. Lung adenocarcinomas are identified in most patients when in an advanced stage, and the malignant cells have already extended into distant organs. These patients are not acceptable for invasive resection. In order to advance the treatment of patients detected with lung cancer, evolving innovative therapies is needed for the treatment of lung cancer. As matured lung cancer cells are very aggressive and lead to high death rates, the inhibition of their invasion and metastasis may be effective in the treatment of lung cancer.

Apoptosis is a serious physiological process responsible for the homeostatic mechanisms to maintain cell populations in tissues [2]. There is extensive research on apoptotic cell death, because of the close relationship between the mechanism of apoptosis and the effect of anticancer agents [3]. Natural products have been reported to induce apoptosis in cancer cells via activating reactive oxygen species (ROS) [4]. ROS induce oxidative DNA damage followed by a leakage of cytochrome c, and subsequently activate the caspase cascade [5]. Besides, the agitation in the expression level of Bax and Blc-2 proteins is a vital factor in regulating the vulnerability of tumor cells to anticancer agents [6]. Cancer metastasis, a highly organized and sequential progression, is characterized by separation of cells from the primary tumor, proteolysis of the extracellular matrix (ECM), intravasation, and then invasion into new tissue and growth [6,7].

Matrix metalloproteinases (MMPs) are the major proteases that contribute to tumor cell migration, tissue invasion, and metastasis [8]. Of these, MMP-2 and -9 play crucial roles in the process of metastasis [9]. Activation of these enzymes is associated with increased tumor metastasis, which proposes a crucial functional role for these proteases in the metastatic process [10]. Previous studies found a reduction in the activities of the antioxidant enzymes glutathione peroxidase (GPx), superoxide dismutase (SOD), and catalase (CAT) in lymphoma cell induced tumors in mice [11], and these enzymes are considered as markers of malignant transformation [12]. Therefore, anticancer agents with the ability to activate apoptosis and antioxidants and suppress MMPs are effective in cancer therapy.

The potential of natural products in cancer prevention and therapy were well described in the special issue titled “Bioactive natural products in cancer prevention and therapy: progress and promise” [13]. A detailed review analytically summarized the role of various natural compounds for cancer prevention and therapy via modulation of different transcription factors, multiple signal transduction, and apoptotic cascades [14,15]. Hinokitiol, a natural compound found in *Chamaecyparis taiwanensis* and scrapped from the wood of cupressaceous plants, has miscellaneous biological and pharmacological properties. Antiviral, antibacterial, antifungal, antitumor, and insecticidal tendencies are the noted biological properties of hinokitiol [16,17]. This natural compound accomplished noteworthy anti-inflammatory activity in a variety of cells, achieved by a range of mechanisms [18]. Hinokitiol is reported to have lung tumor suppressing abilities without changing the body weight and inducing toxicity in the host [17]. Our previous study showed that hinokitiol inhibits cell migration via reducing MMP-1 expression followed by the suppression of nuclear factor κ B (NF-κB)/mitogen-activated protein kinase (MAPKs) signaling pathways and in vivo tumor nodule formation in melanoma cells [19]. Despite hinokitiol being found to inhibit the growth of cancer cells, its role on the inhibition of lung cancer is still unclear. Therefore, this study aimed to investigate the inhibitory effect and the molecular mechanisms of hinokitiol on A549 cells in vitro. This study may provide evidence that hinokitiol can inhibit the migration of A549 cells, which suggests that this natural compound inhibits adenocarcinoma.

## 2. Results

### 2.1. Cytotoxic Effect of Hinokitiol in A549 Cells

**Figure 1:**
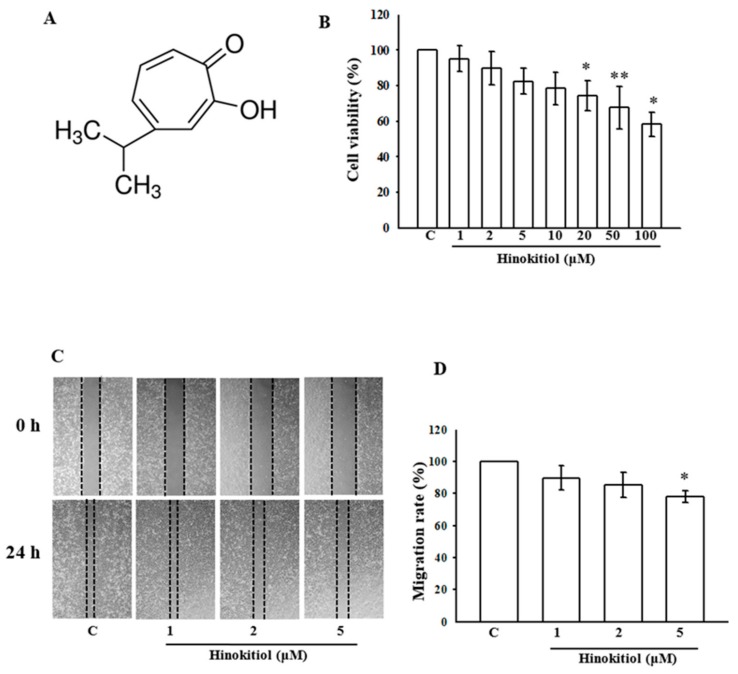
Effects of hinokitiol on the cell viability of the A549 cell line: (**A**) the structure of hinokitiol; (**B**) the viability of A549 cell line during treatment with various concentrations (1~100 µM) of hinokitiol; (**C**,**D**) effects of hinokitiol on A549 cell migration after 24 h of exposure. The figures are representative examples of three independent experiments. * *p* < 0.05 and ** *p* < 0.01 compared with untreated A549 cells.

The chemical structure of hinokitiol is shown in Figure 1A. The cytotoxic effect of hinokitiol on human lung adenocarcinoma A549 cells is demonstrated in Figure 1B. The figure demonstrates that the treatment of more than 20 µM hinokitiol (20–100 µM) for 24 h considerably decreased the viability of A549 cells. The data indicate that treatment with hinokitiol at doses of less than 20 µM (i.e., 1–10 µM) for 24 h does not cause cytotoxicity of A549 cells. Thus, we chose the concentration of 1–5 µM for the current study.

### 2.2. Hinokitiol Inhibits the Migration of A549 Cells

Since the higher concentration of hinokitiol seems toxic, it is obligatory to investigate the inhibitory effect of non-toxic doses of hinokitiol on the migration of A549 cells. After incubation with different concentrations (1–5 µM) of hinokitiol for 24 h, we found the high dose of 5 µM suppresses the migration of A549 cells to the denuded zone (Figure 1C). These results demonstrate that hinokitiol inhibited the migration of A549 cells (Figure 1D).

### 2.3. Effects of Hinokitiol on Caspase Signaling Pathway Activation

**Figure 2:**
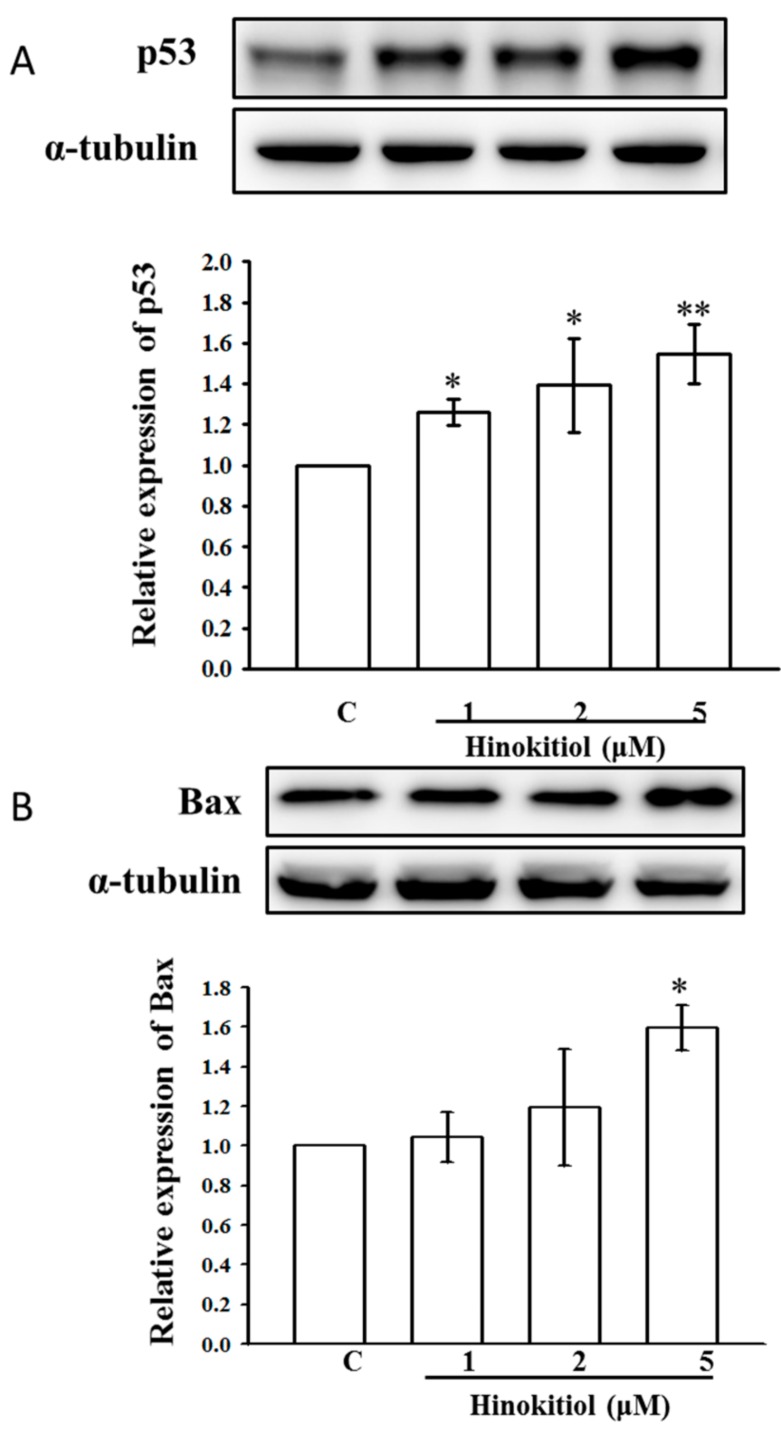
Effects of hinokitiol on phosphorylation of p53 and Bax in A549 cells. A549 adenocarcinoma (5 × 10^4^ cells/well) cells were treated with different concentrations (1–5 µM) of hinokitiol for 30 min. The phosphorylated p53 and Bax proteins in the cell lysate were assayed by Western blotting. Effects of hinokitiol on phosphorylation of p53 (**A**) and Bax (**B**) in A549 adenocarcinoma cells. α-tubulin was used as an internal control. The figures are representative examples of three independent experiments. Data are shown as the mean ± standard errors of the means (SEM) of three independent experiments. * *p* < 0.05, and ** *p* < 0.01 compared with untreated A549 cells.

**Figure 3:**
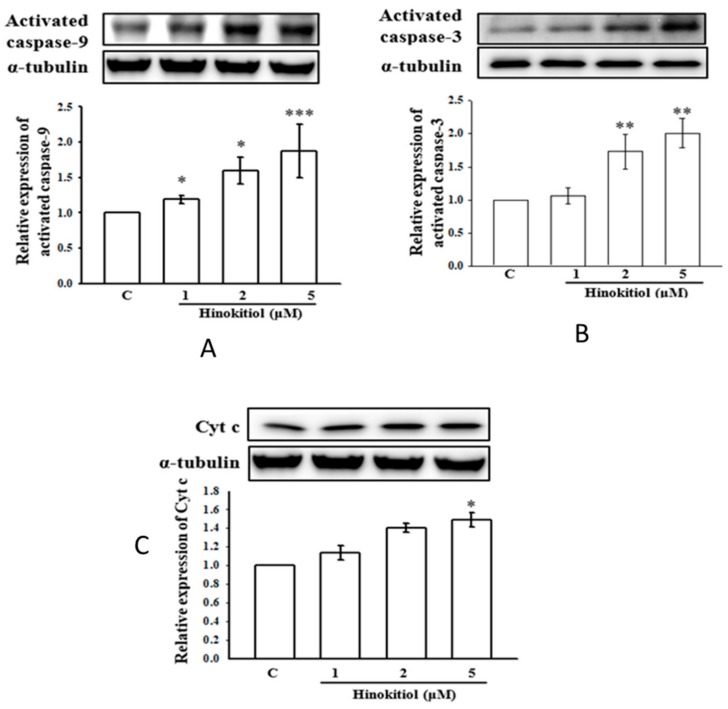
Effect of hinokitiol on caspases activation and cytochrome c release. (**A**,**B**) Relative concentration-dependent activation of activated caspase-9 and -3 in A549 cells treated with various concentrations of hinokitil. Caspase-9 (**A**) was activated in a concentration (1–5 μM) dependent manner, whereas caspase-3 (**B**) was induced significantly only at 2 and 5 μM. (**C**) Meanwhile, cytochrome c was released at the higher concentration (5 μM) of hinokitiol treatment. The data represent the means ± SEM of three independent experiments. * *p* < 0.05, ** *p* < 0.01, and *** *p* < 0.001 compared with untreated A549 cells.

To investigate the molecular mechanisms of hinokitiol-mediated apoptosis in A549 cells, activated caspases-9 and -3 were analyzed by Western blot assay. Studies have proposed that caspases are the main enzymes that regulate apoptosis in tissues or cells. Any stimulatory agents that induce apoptosis were found to activate the effector caspases including caspase-9, caspase-3, and caspase-7 [20]. Similarly, treatment of A549 cells with hinokitiol prominently increases the p53 and Bax protein level (Figure 2A,B). Next, the activated caspase-9 and -3 were enhanced upon hinokitiol administration in a dose-dependent manner (Figure 3A,B). Subsequently, cytochrome c (Cyto-c) was up-regulated, inducing apoptosis in A549 cancer cells (Figure 3C).

### 2.4. Hinokitiol Inhibits MMP-9 and MMP-2 Expression

**Figure 4:**
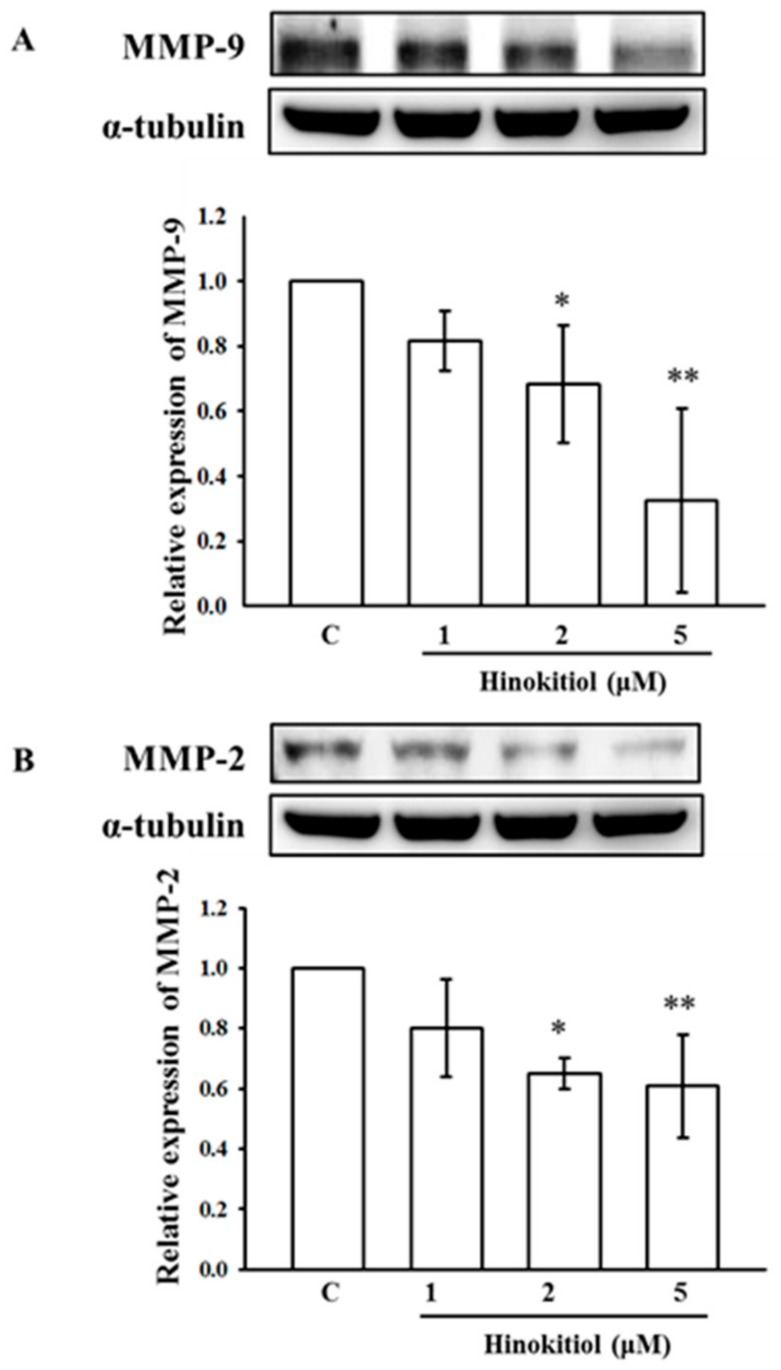
Effects of hinokitiol on matrix metalloproteinases (MMP)-9 and MMP-2 expression in A549 cell line. A549 cells were treated with different concentrations of hinokitiol (1–5 µM) in serum-free media for 24 h. After the treatment periods, cell lysates were collected to detect the expression of MMP-9 (**A**) and MMP-2 (**B**) by using Western blotting. The figures are representative examples of three independent experiments. * *p* < 0.05 and ** *p* < 0.01 compared with untreated A549 cells.

MMP-9 and MMP-2 are recognized as playing a vital role in cancer cell invasion and metastasis among the MMP family. Figure 4A,B exemplifies the expression of MMP-9 and MMP-2 in untreated and hinokitiol treated A549 cells. The results displayed a marked increase in MMP-9 and MMP-2 in untreated A549 cells; this may be due to ROS elevation that triggers MMPs during the rigorous angiogenesis to interrupt the extracellular matrix (ECM). Nevertheless, when cells were treated with hinokitiol, the intracellular protein levels of MMP-9 and MMP-2 were significantly reduced at concentrations of 2 and 5 µM.

### 2.5. Hinokitiol Enhances Antioxidants Enzymes in A549 Cells

**Figure 5:**
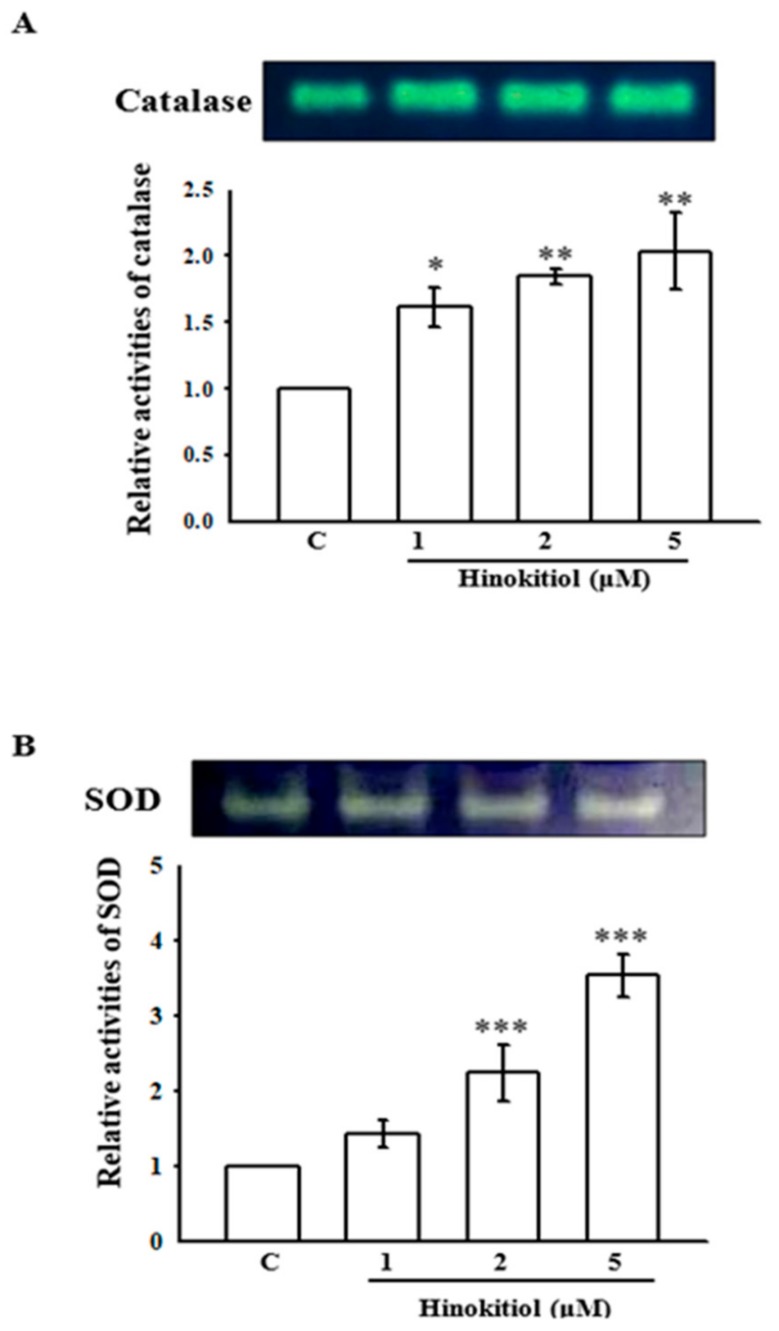
Effects of hinokitiol on catalase (CAT) and superoxide dismutase (SOD) activity in A549 cell line. A549 cells were treated with different concentrations of hinokitiol (1–5 µM) in serum-free media for 24 h. Cell lysates were obtained for the assay of CAT (**A**) and SOD (**B**) activities by using native-PAGE. The figures are representative examples of three independent experiments. * *p* < 0.05, ** *p* < 0.01, and *** *p* < 0.001 compared with untreated A549 cells.

Some studies have shown decreased antioxidant activities in tumors [21,22]. Changes in antioxidant enzymes are associated with the metastatic progression of cancer [23]. In this study, Figure 5A,B reveal the substrate gel activities of catalase (CAT) and superoxide dismutase (SOD) in untreated and hinokitiol treated A549 cells. The activities of CAT and SOD were decreased in untreated A549 cells. Conversely, a concentration-dependent elevation of CAT was observed in hinokitiol treated cells; the highest increase was noticed in 5 µM hinokitiol treated cells (Figure 5A). Likewise, a significant increase in the activity of SOD was also found in 2 and 5 µM hinokitiol-treated cells (Figure 5B). These results propose that hinokitiol can abolish cancer cells through the up-regulation of antioxidant enzymes CAT and SOD.

## 3. Discussion

Chemotherapy with anticancer drugs is more valuable for treating different tumors, mostly in end-stage cancer patients. Nevertheless, the therapeutic methods may encounter drug resistance, as cancer cells can have a diversity of molecular mechanisms to attempt to stay alive by fighting the therapeutic drugs. Hinokitiol is a natural monoterpenoid originally extracted from Taiwanese hinoki, and this compound induces apoptosis in cancer cells via a caspase 3-dependent pathway or through cell cycle arrest [24]. Previous studies indicated that hinokitiol could work as a novel anti-cancer compound. The results of the present study reveal that hinokitiol holds anticancer effects against adenocarcinoma A549 cells via induction of tumor suppressor proteins p53 and Bax and apoptotic markers (activated caspase-9 and -3, cytochrome c), down regulation of MMPs-9 and MMP-2, and up regulation of antioxidants enzymes catalase (CAT) and superoxide dismutase (SOD). This study shows treatment with hinokitiol at doses of less than 20 µM (i.e., 1–10 µM) for 24 h does not cause cytotoxicity in A549 cells. Our previous study found that hinokitiol at the concentrations of 1, 2, and 5 μM did not affect the viability of lymphocytes after treatment for 24  h [25]. Another study from our group also established that hinokitiol (1, 2, 10, and 50 μM) incubated with platelets for 20 min did not significantly increase lactate dehydrogenase (LDH) activity; the study indicated that hinokitiol did not affect platelet permeability or induce platelet cytolysis [26]. These findings clearly show that hinokitiol has no acute cytotoxic effects, as well, it does not induce cytotoxicity in normal cells.

Apoptosis is an important physiological process that arises in cells during growth and normal cellular development [27]. Several cellular signals induce apoptosis and change mitochondrial permeability, resulting in a cascade of actions, such as the release of apoptosis activators from mitochondria [28]. Bax, a pro-apoptotic protein, has been shown to be involved in the cytochrome c release from mitochondria to cytosol via dimerization and translocation to the outer mitochondrial membrane [29]. Moreover, the tumor suppressor p53 induces the cell cycle and apoptosis, which supports genome stability and integrity by responding to cellular stress and DNA damage [30]. The increased levels of p53 inhibit the cyclin expression that is vital for transitioning from G1 into S phase. In this study, p53 and Bax were found to be activated upon hinokitiol administration; this may induce apoptosis. Caspase activation is involved in an energy-dependent cascade of molecular events towards apoptosis. Two major groups of caspases have been recognized to be associated in apoptosis pathways, including executioner caspases-3, -6, -7 and initiator caspases-2, -8, -9, -10 [31]. Initiator caspases-9 and -8 trigger intrinsic and extrinsic pathways, respectively. A previous study reported that activation of these caspases consequently induces executioner caspase-3 [32]. In the present study, we investigated whether hinokitiol triggers the caspases in A549 cells when treated with different concentrations. The expression of caspase-9 and -3 were significantly elevated in hinokitiol treated cells. Our results indicated that induced apoptosis of A549 cells by hinokitiol was mediated through caspase-9 and -3 activation.

Pericellular proteolysis of the extracellular matrix (ECM) is essential for cell projection during cell invasion. The proteolytic degradation of ECM is facilitated by extracellular proteases, such as MMPs, and is required for cancer cell invasion. Among these, MMP-9 and MMP-2 play a critical role in the progression of lung cancer [33]. The inhibition of expression of these MMPs is a potential target for the prevention of the metastasis of cancer [34]. Numerous earlier studies have also established that natural compounds such as quercetin [35], baicalein [36], and gallic acid [37] suppress MMPs to exert their anticancer activity. Consistent with these reports, our study demonstrated that anticancer effects of hinokitiol are associated with a decline in MMP-9 and MMP-2 expression in A549 cells.

Antioxidant systems, which includes SOD, CAT, and glutathione-dependent enzymes are well recognized in lung tissues [38]. SOD enzymes comprise intracellular manganese (Mn)-SOD, copper-zinc (CuZn)-SOD, and an extracellular SOD that occurs in epithelial lining fluid and blood vessels [39]. SOD enzymes convert superoxide anions to H_2_O_2_, and H_2_O_2_ is further converted to water and oxygen by CAT [40]. Previous studies have found reduced antioxidant activity in lung cancers [20,41]. Another study showed reduced catalase activity in lung cancer due to its protein and mitochondrial RNA (mRNA) reduction in tumor cells [42]. Though studies have shown increased antioxidant activities in tumor cells or in individuals with lung cancer [43], others have found the reduction of antioxidant activities in lung tumors [20]. In this study, the antioxidant enzymes CAT and SOD were increased in hinokitiol treated A549 cells.

## 4. Materials and Methods

### 4.1. Materials

Hinikitiol with more than 90% purity was purchased from Sigma (St. Louis, MO, USA). Sodium dodecylsulfate (SDS), phenylmethylsulfonyl fluoride (PMSF), leupeptin, aprotinin, sodium fluoride, sodium orthovanadate, sodium pyrophosphate, diethyl pyrocarbonate (DEPC), bovine serum albumin (BSA), potassium ferricyanide, ferric chloride, nitroblue tetrazolium (NBT), and riboflavin were all purchased from Sigma-Aldrich (St. Louis, MO, USA). An antibody against MMP-9 was purchased from LabVision/NeoMarkers (Fremont, CA, USA). Antibodies against MMP-2 and active caspase-9 and -3 were purchased from BioVision (Mountain View, CA, USA). Anti-mouse and anti-rabbit immunoglobulin G-conjugated horseradish peroxidase (HRP) was purchased from Amersham Biosciences (Sunnyvale, CA, USA) and/or Jackson-Immuno Research (West Grove, PA, USA). The Hybond-P polyvinylidene difluoride (PVDF) membrane, and enhanced chemiluminescence (ECL) Western blotting detection reagents and analysis systems were obtained from Amersham (Buckinghamshire, UK).

### 4.2. Cell Cultivation and Hinokitiol Treatment

Human adenocarcinoma A549 cells were obtained from American Type Culture Collection (Manassas, VA, USA). A549 cells were cultured in RPMI1640 medium contained with 3.65 mM l-glutamine, 90 units/mL penicillin, 90 μg/mL streptomycin, 18 mM HEPES, 23.57 mM NaHCO_3_, and 10% heat-inactivated fetal bovine serum (FBS) at 37 °C in humidified air with 5% CO_2_. In this study, A549 cells were seeded at 5 × 10^4^ per well and cultured until 90% confluent. After 24 h, cells were transferred into serum-free media. Twenty-four hours after changing to serum-free media, cells were treated with hinokitiol (1–5 μM) for another 24 h. At the end of the incubation period, cell supernatants were collected and stored at −80 °C for the Western blot assay.

### 4.3. Cell Viability

The 3-(4,5-dimethylthiazol-2-yl)-2,5-diphenyltetrazolium bromide (MTT) method was used to detect the cell viability. Briefly, 5 × 10^4^ cells were seeded in a 96-well plate containing RPMI1640 medium with 10% fetal bovine serum. After the required confluence was reached, cells were treated with various concentrations of hinokitiol (1–100 µM) for 24 h in a 5% CO_2_ incubator at 37 °C. At 22 h, the medium was changed to fresh medium having 0.5 mg/mL MTT. After 2 h incubation, the dark blue MTT formazan crystals formed in intact cells were solubilized in dimethyl sulfoxide (DMSO), and the absorbance was measured at 550 nm in a spectrophotometer. The percent cell viability was calculated using the following formula:

Percentage cell viability = (absorbance of the experiment samples/absorbance of the control) × 100%

### 4.4. Wound Healing Migration Assay

According to a previously described study, the wound healing migration assay was executed [44]. In brief, A549 cells were seeded in 12-well plates to achieve the required growth. The monolayer culture was then scrape-wounded with a sterile micropipette tip to create a denuded zone with constant width. After removing the cellular debris using PBS, cells were treated with various concentrations (1–5 µM) of hinokitiol for 24 h. A549 cell migration to the wounded region was monitored by photographing at 0 and 24 h using an Image pro Express, version 6.0.0.319 for Windows XP/Professional (Media Cybernetics Inc., Bethesda, MD, USA). To quantify cell migration, images of the initial wounded monolayers were equated to the corresponding pictures of cells at later time points. Migrated cells in each of five random fields were counted.

### 4.5. Western Blotting 

Cells with required confluence were pre-incubated with 1–5 µM hinokitiol for 24 h for MMPs-9 and MMP-2, activated caspases-9 and -3, and cytochrome c and 30 min for p53 and Bax. After the experimental periods, the proteins were extracted with 60 µL lysis buffer. Samples containing 50 µg of protein were separated by 10% SDS–PAGE, and the proteins were electrotransferred to the PVDF membranes using a Bio-Rad semi dry transfer unit (Hercules, CA, USA). The membranes were blocked with 5% (*w*/*v*) non-fat milk in TBST (10 mM Tris-base, 100 mM NaCl, and 0.01% Tween 20) for 40 min, and blotted with the various primary antibodies. Subsequently, the membranes were incubated with an appropriate secondary antibody (horseradish peroxidase-conjugated goat anti-mouse or anti-rabbit IgG). The immunoreactive bands were visualized with enhanced chemiluminescent reagents (ECL, Amersham, UK).

### 4.6. Non-Denaturing Polyacrylamide Gel Electrophoresis (Native PAGE)

The activity of the antioxidant enzymes CAT and SOD was detected by using native PAGE. For this, the buffers and samples were not heated in the absence of SDS before electrophoresis. The PAGE was run based on the equal amounts of 50 µg protein in an 8% gel for CAT and 10% gel for SOD. The electrophoretic gel was run at 4 °C with a constant power supply of 80 V for stacking gel and 100 V for separating gel.

#### 4.6.1. CAT Activity Staining

The activity of the CAT enzyme was identified according to the method described by Woodbury et al. [45]. In this, the gel was incubated in 5 mM H_2_O_2_ solution for 10 min, washed with deionized water, and stained with a reaction mixture containing 1% potassium ferricyanide (*w*/*v*) and 1% ferric chloride. The appearance of a yellow band on a dark green background is considered to be indicative of the CAT enzyme. The reaction was ended by adding water, and the gel was photographed.

#### 4.6.2. SOD Activity Staining

SOD activity was identified according to the method described by Beauchamp and Fridovich [46]. The gel was incubated in 50 mM Tris-HCl buffer (pH 8) containing 10 mg nitroblue tetrazolium (NBT), 1 mg ethylene diamine tetra acetic acid (EDTA), and 2 mg riboflavin (50 mL final volume) and reserved in a dark place for 30 min. The gel was then taken to an illuminated light box to detect the area of SOD activity, which looked like a clear zone on a bluish-violet background.

### 4.7. Statistical Analysis

The results are expressed as the mean ± standard errors of the means (SEM) and are accompanied by the number of observations. For analysis of the data, a one-way analysis of variance (ANOVA) test was performed using the Sigma Stat v3.5 software (SAS Inc., Cary, NC, USA). When group comparisons displayed a significant difference, the Student-Newman-Keuls test was applied. *p*-value < 0.05 was considered statistically significant.

## 5. Conclusions

This study shows the inhibitory effect of hinokitiol on the migration of human adenocarcinoma A549 cells. The anticancer potential of hinokitiol is supported by the evidence provided in the present study, including, upregulation of Bax/p53, increase in the level of cytochrome c, and activation of caspases-9/-3 and antioxidant enzymes CAT and SOD. Moreover, we found the inhibitory effect of hinokitiol on the A549 cell migration was accompanied by downregulation of MMPs-9 and MMP-2. These results may illustrate a new therapeutic value of hinokitiol in anticancer therapy.

## References

[1] Shivapurkar N., Reddy J., Chaudhary P.M., Gazdar A.F. (2003). Apoptosis and lung cancer: A review. J. Cell. Biochem..

[2] Kim S.S., Cho H.J., Kang J.Y., Kang H.K., Yoo T.K. (2013). Inhibition of androgen receptor expression with small interfering RNA enhances cancer cell apoptosis by suppressing survival factors in androgen insensitive, late stage LNCaP cells. Sci. World J..

[3] Ocker M., Höpfner M. (2012). Apoptosis-modulating drugs for improved cancer therapy. Eur. Surg. Res..

[4] Moghadamtousi S.Z., Goh B.H., Chan C.K., Shabab T., Kadir H.A. (2013). Biological activities and phytochemicals of *Swietenia macrophylla* king. Molecules.

[5] Simon H.U., Haj-Yehia A., Levi-Schaffer F. (2000). Role of reactive oxygen species (ROS) in apoptosis induction. Apoptosis.

[6] Martinou J.C., Youle R.J. (2011). Mitochondria in apoptosis: Bcl-2 family members and mitochondrial dynamics. Dev. Cell.

[7] Yang F., Jove V., Chang S., Hedvat M., Liu L., Buettner R., Tian Y., Scuto A., Wen W., Yip M.L.R. (2012). Bortezomib induces apoptosis and growth suppression in human medulloblastoma cells, associated with inhibition of AKT and NF-kB signaling, and synergizes with an ERK inhibitor. Cancer Biol. Ther..

[8] Itoh Y., Nagase H. (2002). Matrix metalloproteinases in cancer. Essays Biochem..

[9] Bernhard E.J., Gruber S.B., Muschel R.J. (1994). Direct evidence linking expression of matrix metalloproteinase 9 (92-kDa gelatinase/collagenase) to the metastatic phenotype in transformed rat embryo cells. Proc. Natl. Acad. Sci. USA.

[10] Mook O.R., Frederiks W.M., Van Noorden C.J. (2004). The role of gelatinases in colorectal cancer progression and metastasis. Biochim. Biophys. Acta.

[11] Loganayaki M., Manian S. (2012). Antitumor activity of the methanolic extract of *Ammannia baccifera* L. against Dalton’s ascites lymphoma induced ascetic and solid tumors in mice. J. Ethnopharmacol..

[12] Kavitha K., Manoharan S. (2006). Anticarcinogenic and antilipidperoxidative effects of *Tephrosia purpurea* (Linn) pers. In 7, 12-dimethyl benz (a) anthracene (DMBA) induced hamster buccal pouch carcinoma. Ind. J. Pharmacol..

[13] Bishayee A., Sethi G. (2016). Bioactive natural products in cancer prevention and therapy: Progress and promise. Semin. Cancer Biol..

[14] Shanmugam M.K., Lee J.H., Chai E.Z., Kanchi M.M., Kar S., Arfuso F., Dharmarajan A., Kumar A.P., Ramar P.S., Looi C.Y. (2016). Cancer prevention and therapy through the modulation of transcription factors by bioactive natural compounds. Semin. Cancer Biol..

[15] Shanmugam M.K., Kannaiyan R., Sethi G. (2011). Targeting cell signaling and apoptotic pathways by dietary agents: Role in the prevention and treatment of cancer. Nutr. Cancer.

[16] Baba T., Nakano H., Tamai K., Sawamura D., Hanada K., Hashimoto I., Arima Y. (1998). Inhibitory effect of beta-thujaplicin on ultraviolet B-induced apoptosis in mouse keratinocytes. J. Investig. Dermatol..

[17] Li L.H., Wu P., Lee J.Y., Li P.R., Hsieh W.Y., Ho C.C., Ho C.L., Chen W.J., Wang C.C., Yen M.Y. (2014). Hinokitiol induces DNA damage and autophagy followed by cell cycle arrest and senescence in gefitinib-resistant lung adenocarcinoma cells. PLoS ONE.

[18] Shih Y.H., Lin D.J., Chang K.H., Hsia S.M., Ko S.Y., Lee S.Y., Hsue S.S., Wang T.H., Chen Y.L., Shieh T.M. (2014). Evaluation physical characteristics and comparison antimicrobial and anti-inflammation potentials of dental root canal sealers containing hinokitiol in vitro. PLoS ONE.

[19] Huang C.H., Lu S.H., Chang C.C., Thomas P.A., Jayakumar T., Sheu J.R. (2015). Hinokitiol, a tropolone derivative, inhibits mouse melanoma (B16-F10) cell migration and in vivo tumor formation. Eur. J. Pharmacol..

[20] Lavrik I.N., Golks A., Krammer P.H. (2005). Caspases: Pharmacological manipulation of cell death. J. Clin. Investg..

[21] Guner G., Islekel H., Oto O., Hazan E., Acikel U. (1996). Evaluation of some antioxidant enzymes in lung carcinoma tissue. Cancer Lett..

[22] Coursin D.B., Cihla H.P., Sempf J., Oberley T.D., Oberley L.W. (1996). An immunohistochemical analysis of antioxidant and glutathione s-transferase enzyme levels in normal and neoplastic human lung. Histol. Histopathol..

[23] Lewis A., Du J., Liu J., Ritchie J.M., Oberley L.W., Cullen J.J. (2005). Metastatic progression of pancreatic cancer: Changes in antioxidant enzymes and cell growth. Clin. Exp. Metast..

[24] Lee Y.S., Choi K.M., Kim W., Jeon Y.S., Lee Y.M., Hong J.T., Yun Y.P., Yoo H.S. (2013). Hinokitiol inhibits cell growth through induction of S-phase arrest and apoptosis in human colon cancer cells and suppresses tumor growth in a mouse xenograft experiment. J. Nat. Prod..

[25] Chung C.L., Leung K.W., Lu W.J., Yen T.L., He C.F., Sheu J.R., Lin K.H., Lien L.M. (2015). Hinokitiol negatively regulates immune responses through cell cycle arrest in concanavalin A-activated lymphocytes. Evid. Based Complement. Altern. Med..

[26] Lin K.H., Kuo J.R., Lu W.J., Chung C.L., Chou D.S., Huang S.Y., Lee H.C., Sheu J.R. (2013). Hinokitiol inhibits platelet activation ex vivo and thrombus formation in vivo. Biochem. Pharmacol..

[27] Abedin M.J., Wang D., McDonnell M.A., Lehmann U., Kelekar A. (2007). Autophagy delays apoptotic death in breast cancer cells following DNA damage. Cell Death Differ..

[28] Xiong X., Wu M., Zhang H., Li J., Lu B., Guo Y., Zhou T., Guo H., Peng R., Li X. (2015). Atg5 siRNA inhibits autophagy and enhances norcantharidin-induced apoptosis in hepatocellular carcinoma. Int. J. Oncol..

[29] Zamzami N., Kroemer G. (2001). The mitochondrion in apoptosis: How Pandora’s box opens. Nat. Rev. Mol. Cell Biol..

[30] Baldwin A.S. (2001). Control of oncogenesis and cancer therapy resistance by the transcription factor NF-κB. J. Clin. Investg..

[31] Hill M.M., Adrain C., Duriez P.J., Creagh E.M., Martin S.J. (2004). Analysis of the composition, assembly kinetics and activity of native Apaf-1 apoptosomes. EMBO J..

[32] Elmore S. (2007). Apoptosis: A review of programmed cell death. Toxicol. Pathol..

[33] Bodey B., Bodey B., Groger A.M., Siegel S.E., Kaiser H.E. (2001). Invasion and metastasis: The expression and significance of matrix metalloproteinases in carcinomas of the lung. In Vivo.

[34] Hrabec E., Strek M., Nowak D., Greger J., Suwalski M., Hrabec Z. (2002). Activity of type IV collagenases (MMP-2 and MMP-9) in primary pulmonary carcinomas: A quantitative analysis. J. Cancer Res. Clin. Oncol..

[35] Lai W.W., Hsu S.C., Chueh F.S., Chen Y.Y., Yang J.S., Lin J.P., Lien J.C., Tsai C.H., Chung J.G. (2013). Quercetin inhibits migration and invasion of SAS human oral cancer cells through inhibition of NF-κB and matrix metalloproteinase-2/-9 signaling pathways. Anticancer Res..

[36] Chandrashekar N., Selvamani A., Subramanian R., Pandi A., Thiruvengadam D. (2012). Baicalein inhibits pulmonary carcinogenesis-associated inflammation and interferes with COX-2, MMP-2 and MMP-9 expressions in-vivo. Toxicol. Appl. Pharmacol..

[37] Subramanian V., Venkatesan B., Tumala A., Vellaichamy E. (2014). Topical application of Gallic acid suppresses the 7, 12-DMBA/Croton oil induced two-step skin carcinogenesis by modulating anti-oxidants and MMP-2/MMP-9 in Swiss albino mice. Food Chem. Toxicol..

[38] Seidman M.D., Quirk W.S., Shirwany N.A. (1999). Reactive oxygen metabolites, antioxidants and head and neck cancer. Head Neck.

[39] Erzurum S.C., Danel C., Gillissen A., Chu C.S., Trapnell B.C., Crystal R.G. (1993). In vivo antioxidant gene expression in human airway epithelium of normal individuals exposed to 100% O_2_. J. Appl. Physiol..

[40] Putnam C.D., Arvai A.S., Bourne Y., Tainer J.A. (2000). Active and inhibited human catalase structures: Ligand and NADPH binding and catalytic mechanism. J. Mol. Biol..

[41] Crawford E.L., Khuder S.A., Durham S.J., Frampton M., Utell M., Thilly W.G., Weaver D.A., Ferencak W.J., Jennings C.A., Hammersley J.R. (2000). Normal bronchial epithelial cell expression of glutathione transferase P1, glutathione transferase M3, and glutathione peroxidase is low in subjects with bronchogenic carcinoma. Cancer Res..

[42] Ho C.J., Zheng S., Comhair S.A.A., Farver C., Erzurum S.C. (2001). Differential expression of manganese superoxide dismutase and catalase in lung cancer. Cancer Res..

[43] Melloni B., Lefebvre M., Bonnaud F., Vergnenegre A., Grossin L., Rigaud M., Cantin A. (1996). Antioxidant activity in bronchoalveolar lavage fluid from individuals with lung cancer. Am. J. Respir. Crit. Care Med..

[44] Lu M.K., Shih Y.W., Chang T.T., Fang L.H., Huang H.C., Chen P.S. (2010). α-Solanine inhibits human melanoma cell migration and invasion by reducing matrix metalloproteinase-2/9 activities. Biol. Pharm. Bull..

[45] Woodbury W., Speacer A.K., Stahman M.A. (1971). An improved procedure using ferricyanide for detecting catalase isozymes. Anal. Biochem..

[46] Beauchamp C., Fridovich I. (1971). Superoxide dismutase: Improved assays and an assay applicable to acrylamide gels. Anal. Biochem..

